# Prognostic Indicators of Low Back Pain in Primary Care: Five-Year Prospective Study

**DOI:** 10.1016/j.jpain.2013.03.013

**Published:** 2013-08

**Authors:** Paul Campbell, Nadine E. Foster, Elaine Thomas, Kate M. Dunn

**Affiliations:** Arthritis Research UK Primary Care Centre, Keele University, Keele, Staffordshire, United Kingdom

**Keywords:** Prognosis, low back pain, cohort, longitudinal, prospective

## Abstract

Back pain is common and many people experience long-term problems, yet little is known about what prognostic factors predict long-term outcomes. This study's objective was to determine which factors predict short- and long-term outcomes in primary care consulters with low back pain (LBP). Analysis was carried out on 488 patients who had consulted their physician about LBP. Patients were followed up at 6 months and 5 years. Clinically significant LBP at follow-up was defined as a score of 2, 3, or 4 on the Chronic Pain Grade, indicating substantial pain and disability. Cox regression was used to estimate relative risks (RRs) with 95% confidence intervals (CIs) on 32 potential predictive factors, organized into domains (demographic, physical, psychological, and occupational). Baseline pain intensity conferred a 12% increase in risk (RR = 1.12, 95% CI = 1.03–1.20), and patients' belief that their LBP would persist conferred a 4% increase in risk (RR = 1.04, 95% CI = 1.01–1.07) for poor outcome at 6 months. Outcome at 5 years was best predicted by a model with the same factors as in the 6-month model: pain intensity increased risk by 9% (RR = 1.09, 95% CI = .997–1.20), and a belief that their LBP would persist increased risk by 6% (RR = 1.06, 95% CI = 1.03–1.09). Both predictors have the potential to be targets for clinical intervention.

**Perspective:**

Few studies have investigated factors that predict long-term back pain. This study has shown that pain intensity experienced during a period of primary care consultation, and patients' perception about whether their back pain will persist, were significant predictors of poor outcome at 6 months and at 5 years.

The prevalence of low back pain (LBP) is substantial, with population estimates of 50 to 70% over a lifetime.^21^, ^30^ Up to half the people with LBP seek health care for their pain.^21^ Evidence highlights that many people with LBP do not have single episodes but often experience long-term pain with significant recurrence and fluctuations.^15^, ^17^, ^21^, ^31^ This results in considerable costs for health care and society.^25^

One important area of focus within LBP research is the identification of key prognostic factors.^15^ There is a diverse range of prognostic factors in relation to LBP: demographics such as educational status, age, and gender,^18^ physical factors such as the level of pain intensity and disability perceived by the patient,^24^ psychological factors such as depression and anxiety^23^ and pain-specific concepts such as fear avoidance, catastrophizing, and illness perceptions,^4^, ^10^, ^12^ and occupational factors such as employment status.^8^, ^12^ Importantly, these factors can characterize groups of people at higher risk of persistent pain and disability, and they highlight potentially modifiable factors to target in clinical interventions (eg, psychological therapies and occupational interventions).^15^, ^24^ However, most prognostic studies of LBP have considered follow-up periods of 1 year or less (see reviews^17^, ^24^). For example, of the 32 studies on back or spinal pain included in a review by Mallen and colleagues,^24^ only 3 had follow-up periods longer than 1 year. This is problematic, as potential prognostic factors could differ depending on the time scale.^4^, ^6^, ^15^ For example, one study (Burton et al^4^) followed up patients attending private group osteopathic practices. They tested factors that were associated with disability at 1 year and at 4 years and report that fear avoidance, passive coping, and catastrophizing were significant at 1 year, but depression and pain intensity were significant at 4 years. They suggested that initial fear avoidance, catastrophizing, and passive coping possibly give way to depression in the long term. However, another recent study^12^ considered differences in prognostic factors between primary care patients with acute/subacute pain (defined as pain duration of less than 3 months prior to baseline assessment) and those with chronic pain (defined as pain duration of more than 3 months prior to baseline assessment) at baseline. They reported no differences in prognostic factors between these groups in the prediction of disability 12 months later. This clearly shows that further study is required to understand the potential for differences in prognostic markers dependent on time. Indeed possible differences in prognostic factors over time may be a reason why current interventions for LBP show low sustainability of treatment effect over the long term.^1^ We need to better characterize factors that independently predict short- and long-term outcome of patients with LBP in order to inform and test treatments that target different prognostic groups.

The aim of this study is to investigate, in patients with LBP consulting in a primary care setting, which prognostic factors predict poor pain and disability outcomes 5 years later and to compare these with predictors of earlier short-term outcomes at 6-month follow-up in the same cohort.

## Methods

### Design and Setting

Participants in a large prospective cohort study of persons visiting their primary care physician about LBP were mailed questionnaires soon after their healthcare visit (baseline) and again 6 months and 5 years later. The population for this analysis were responders to the baseline questionnaire (N = 1591) who gave consent to further contact and who responded again at 6 months (n = 810) and 5 years (n = 488).

Ethical approval was given by North Staffordshire and North West Cheshire Research Ethics Committees for all phases of the study.

### Recruitment and Procedure

**Figure 1:**
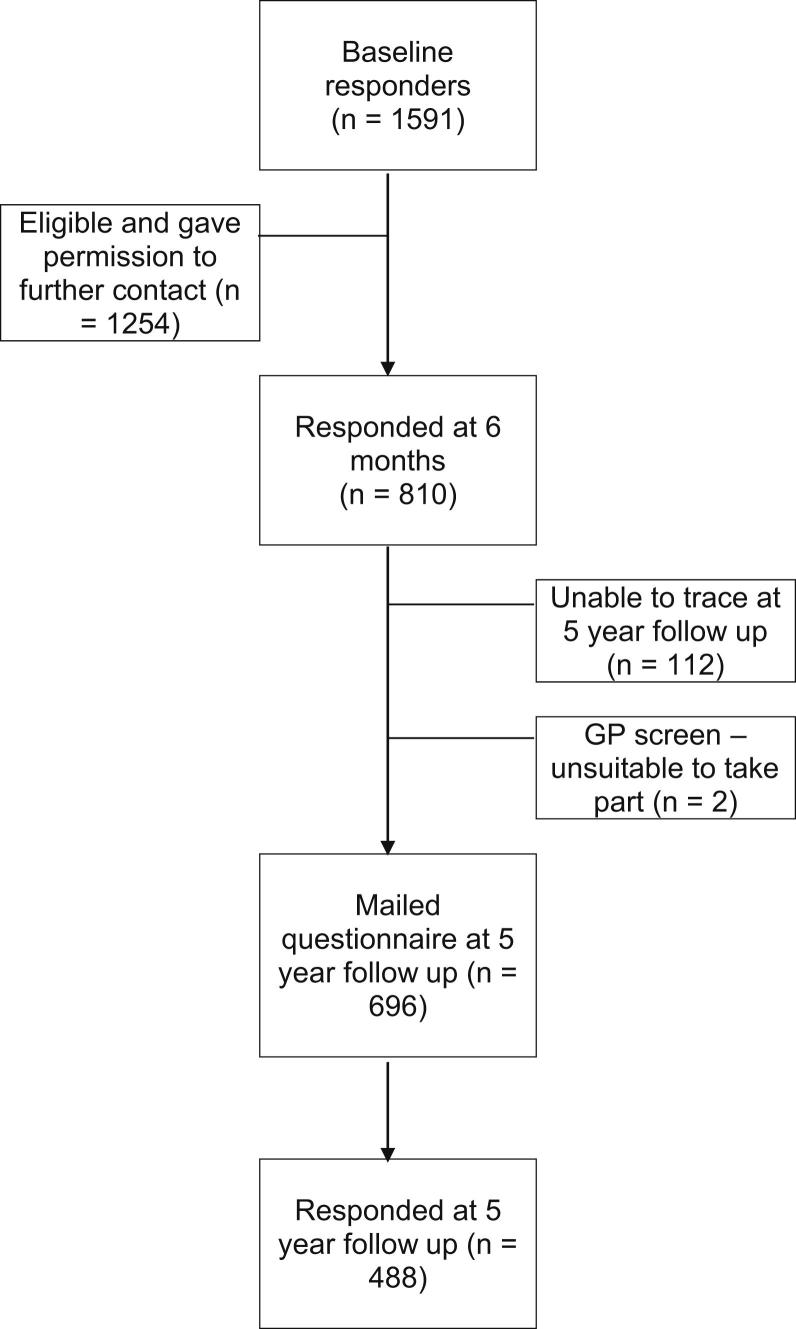
Flow diagram of recruitment.

Patients, aged between 18 and 60 years, who visited their primary care physician about LBP at 8 primary care practices within the North Staffordshire and Cheshire area of England were invited to take part.^10^ Primary care practices are the gateway to the healthcare system within the United Kingdom. The practices cover a range of deprivation areas, and given that more than 95% of the UK population is registered with a primary care practice,^2^ they are representative of the local population. At baseline, eligible participants were identified via computerized primary care records using the “Read Code” system, which is the standard method of coding and recording reasons for contact in UK general practice. All codes relating to LBP were used to identify potential participants, with specific codes for “red flag” diagnoses (cauda equina syndrome, significant trauma, ankylosing spondylitis, cancers) used as exclusion criteria. Quality and validity of the Read Code system within these practices is assessed annually through continual training and feedback to ensure high levels of recording of relevant Read codes during patient healthcare visits.^33^ The target cohort consisted of 1,591 adults who had visited their primary care physician for LBP in the study practices and who responded to the initial baseline questionnaire mailed to them within 2 weeks of their index visit. We have previously shown that this cohort is broadly representative of all patients attending primary care for LBP in these practices^10^ and that the registered populations are broadly representative of a UK population generally. Of the 1,591 back pain patients recruited at baseline, 810 completed and returned their 6-month follow-up questionnaire and 488 responded at 5 years (70% of those eligible). This cohort of 488 responders at 5 years formed the basis for the analyses presented in this paper (see flow diagram Fig 1).

### Measures

#### Outcome Measure

Pain and disability related to LBP were measured at 6 months and at 5 years using the Chronic Pain Grade (CPG).^41^, ^42^ The CPG is a 7-item measure of chronic pain for assessing the patient using 2 dimensions: pain severity (pain at present time and worst and average pain over the previous 6 months) and disability (pain interference, social and employment activity restriction due to pain over the previous 6 months). The measure categorizes outcome of chronic pain and disability into 5 grades: Grade 0 (pain free), Grade 1 (low disability, low pain intensity), Grade 2 (low disability, high pain intensity), Grade 3 (high disability, pain that is moderately limiting), and Grade 4 (high disability, pain that is highly limiting). The CPG has established validity in the UK population^37^ and as a measure of pain over time.^9^ For the purpose of this study, CPG scores were collapsed to form 2 groups, with Grades 0 and 1 forming a group with no or low levels of pain or disability and Grades 2, 3, and 4 forming a clinically significant LBP group, following previous methodology,^11^, ^41^ with the latter being defined as a poor outcome.

### Demographic Predictors

Participants were grouped into 1 of 4 age categories—≤37, 38 to 45, 46 to 52, and ≥53 years—because of the nonlinear relationship of age and LBP^18^; gender was used as a dichotomized variable. Educational status was categorized into 2 groups, education up to the age of 16 and educated beyond the age of 16, and social class was dichotomized^29^ into higher (managerial, professional, intermediate, self-employed) and lower (supervisory, technical, routine occupations), as used previously.^12^

### Physical Predictors at Baseline

LBP disability was assessed using the 24-item Roland Morris Disability Questionnaire (RMDQ).^35^ The RMDQ asks questions on the level of disability associated with LBP on the day of questioning and gives a score from 0 to 24 (a higher score indicates a higher level of disability).

Pain intensity was measured by calculating the mean of 3 numerical rating scales (0–10) for the participant's least and usual LBP over the previous 2 weeks, and their rating of current pain intensity at the time of filling in the questionnaire.^6^ A higher score indicates a higher level of reported pain intensity.

Symptom duration has been shown to be an important prognostic marker for poor outcome.^7^ Pain duration at baseline was measured by asking participants to recall when they had their last “pain-free” month. Two groups were created: 1) pain duration less than 3 years prior to baseline and 2) pain duration more than 3 years prior to baseline. This choice of timeline (duration of 3 years) was based on a recent prognostic study that included the influence of prior to baseline indication of pain duration (ie, how long patient had pain before entering the study) as a risk factor. They reported no differences in outcome (in their case recovery) using the usual 3 months acute-chronic distinction; however, they did report differences on outcome using the above- or below-3-year category.^5^

The presence of leg pain and distal pain (eg, above, below knee) and of upper body pain (shoulder, arm, neck, or head) was also recorded.^5^, ^7^

### Psychological Predictors

The Illness Perception Questionnaire–Revised (IPQ-R) was used to assess illness perceptions.^26^ The IPQ-R has 12 subscales, 8 for illness perceptions (illness identity, consequences, timeline–acute to chronic, timeline–cyclical, illness coherence, treatment control, personal control, emotional representations) and 4 on the causes of LBP (accident/chance, psychological, risk factors, immunity). Higher scores on each subscale of the IPQ-R indicate stronger illness perceptions, with some intersubscale items being reverse scored.

Fear of movement was measured by the Tampa Scale for Kinesiophobia (TSK).^20^ The TSK contains 17 items about a person's fear of movement because of pain, with higher scores indicating a higher level of movement avoidance.

The Coping Strategies Questionnaire 24 (CSQ-24) was used to assess coping styles in participants.^14^ Four scales are used in the questionnaire (catastrophizing, diversion, reinterpretation, cognitive-coping), with higher scores indicating a higher frequency of use of the coping style.

In addition, the baseline questionnaire contained 12 items on behavioral coping in relation to the patient's pain (use of over-the-counter medicine, lying down, creams or sprays, hot and cold packs, massage, lumbar support, walking, swimming, general practitioner-prescribed medication, walking stick, bed rest, exercise). Using guidance from Brown and Nicassio's active and passive coping model,^3^ exploratory factor analysis was performed on these items. Analysis revealed suitability of these items to factor analysis, and a 2-factor “active” and “passive” coping construct was created.^43^

The Pain Self-Efficacy Questionnaire (PSEQ) was used to measure participants' beliefs and confidence in their ability to accomplish activities and engage in situations (eg, doing household chores, being active, getting enjoyment out of things, and leading a normal life) despite their level of pain.^27^ The measure consists of 10 items, each scored by a 6-point Likert-type scale, with a higher score indicating greater self-efficacy.

The Hospital Anxiety and Depression Scale (HADS) was used to measure depressive and anxiety symptoms.^44^ The measure consists of 14 questions and produces a scale from 0 to 21 for depression and anxiety separately, and is applicable to general population samples.

### Occupational Predictors

Four groups were created on the basis of the reports of the respondents current work status: 1) currently employed in normal job, 2) currently employed on reduced duties because of LBP, 3) currently unemployed because of LBP, and 4) currently unemployed because of other reasons.

### Statistical Analysis

Univariate regression was performed on all predictors in relation to poor outcome at 6 month and at 5 years. Cox regression, with a constant time variable,^40^ was used to determine an unadjusted regression coefficient (relative risk [RR]) for each predictor.^22^ All predictors with *P* values < .1 at this stage were retained for multivariable analysis. Prognostic factors were then examined within 4 domains (ie, demographic factors, physical factors, psychological factors, and occupational factors). Domains were chosen to coherently manage the large number of variables within the data set, and to extract the predictor or predictors that best explain the association with poor outcome from the respective domains (ie, the key demographic factors, physical factors, and psychological factors). For example, Pincus et al^32^ has suggested that many psychological variables related to pain overlap conceptually. Therefore, this current approach allowed for mutual adjustment to account for statistical overlap that may be present between variables (intercorrelation) and to identify the significant variable or variables for that domain. All nonsignificant predictors (*P* > .05) in each domain-specific model were removed. The remaining predictors from each domain-specific model were then entered into a final multivariable model with each other for outcomes at 6-month and 5-year follow-up time points, which produced adjusted RRs with 95% confidence intervals (CIs).

Missing data and the impact of nonresponse at baseline and follow-up was analyzed by comparing participants' baseline response on individual prognostic factors with response status at 6 months and 5 years, using summary measures (Appendix 1). Checks for multicollinearity were carried out because of the large number of variables with the potential to have statistical overlap, which can then affect the unique estimates of each variable and lead to instability within the coefficient estimates. Following guidelines for multivariable analysis, each variable was set sequentially as a dependent variable, and all others were set as independent variables using linear regression.^19^ Variance inflation factor (VIF) values were inspected to indicate the regression coefficient influence from all other variables; variables with VIF scores >4 would be considered problematic.

## Results

Baseline characteristics show a mean age of the cohort of 47.4 years, with just over 62% being female. More than half (58.6%) indicated being in education beyond 16 years of age and just over 60% were employed. More than 22% describe experiencing their LBP for more than 3 years, with over 60% describing pain spreading to their legs. Full details for the study participants included in this analysis are presented in Table 1. No variables were found to be highly related (ie, VIF score >4).

**Table 1:** Baseline Characteristics of 488 LBP Patients Seeking Consultation

Baseline Characteristics	Number (%)	Mean (SD)	Interquartile Range
Demographic			
Age (years)		47.4 (9.0)	41–45
Gender (female)	303 (62.1)		
Education (beyond 16)	286 (58.6)		
Social class (low)	184 (37.7)		
Physical			
Pain intensity		3.9 (2.3)	2.3–5.3
RMDQ		8.5 (5.9)	4–13
Pain duration (>3 years)	109 (22.3)		
Leg pain (yes)	299 (61.3)		
Distal pain (yes)	168 (34.4)		
Psychological			
HADS, Anxiety symptoms		8.1 (4.4)	5–11
HADS, Depressive symptoms		6.2 (4.2)	3–9
TSK, Fear of movement		39.0 (6.9)	35–43
CSQ, Catastrophizing		9.2 (7.4)	3–13
CSQ, Diversion		16.0 (8.2)	10–21
CSQ, Reinterpretation		7.5 (6.8)	2–11
CSQ, Cognitive coping		17.0 (6.0)	13–21
IPQR, Symptoms		4.1 (2.3)	2–5
IPQR, Consequences		17.2 (5.5)	13–21
IPQR, Cyclical time		13.0 (3.3)	11–16
IPQR, Emotional representation		16.5 (5.2)	13–20
IPQR, Illness coherence		13.1 (5.0)	10–17
IPQR, Personal control		21.1 (3.7)	18–24
IPQR, Treatment control		17.2 (3.2)	15–20
IPQR, Timeline acute-chronic		19.9 (5.7)	16–24
IPQR, Psychological attributions		11.6 (3.9)	9–14
IPQR, Risk factor attributions		15.0 (4.0)	12–17
IPQR, Immunity dimension		5.2 (1.9)	3–6
IPQR, Accident chance		5.9 (1.9)	5–7
Pain self-efficacy		39.0 (6.9)	29–50
Active behavioral coping		1.2 (.9)	0–2
Passive behavioral coping		2.2 (1.4)	1–3
Occupational			
Employed	294 (60.9)		
Employed, reduced duties due to LBP	74 (15.3)		
Unemployed	78 (16.1)		
Unemployed due to LBP	37 (7.7)		

At 6 months, 47.7% had clinically significant LBP, falling to 36.9% at the 5-year follow-up. The regression models showing the baseline factors predicting clinically significant LBP status at the 6-month and 5-year follow-ups are presented in Table 2.

**Table 2:** Cox Regression Models for the Relationship Between Prognostic Indicators at Baseline and Clinically Significant LBP Group at 6-Month and 5-Year Follow-Up Stages

Prognostic Indicators	Unadjusted RR (95% CI)		Domain Adjustment RR (95% CI)		Final Model RR (95% CI)	
	6-Month	5-Year	6-Month	5-Year	6-Month	5-Year
Demographics						
Age, years (<38 reference)						
38–45	.949 (.64, 1.4)	.959 (.60, 1.5)				
46–52	.949 (.67, 1.4)	.933 (.59, 1.5)				
>52	1.05 (.76, 1.5)	.891 (.57, 1.4)				
Gender (male as reference)	1.32∗ (1.0, 1.8)	1.15 (.84, 1.6)	1.31 (.98, 1.8)			
Education (>16 as reference)	1.36∗ (1.0, 1.8)	1.46∗ (1.1, 2.0)	1.16 (.88, 1.5)	1.28 (.93, 1.8)		
Social class (high as reference)	1.45∗ (1.1, 1.9)	1.54∗ (1.1, 2.1)	1.47∗ (1.1, 1.9)	1.47∗ (1.1, 2.1)	1.14 (.85, 1.5)	1.19 (.86, 1.7)
Pain						
Pain Intensity	1.23∗∗ (1.2, 1.3)	1.24∗∗ (1.2, 1.3)	1.15∗∗ (1.1, 1.2)	1.11∗ (1.0, 1.2)	1.12∗ (1.0, 1.2)	1.09 (1.0, 1.2)
RMDQ	1.08∗∗ (1.1, 1.1)	1.09∗∗ (1.1, 1.1)	1.04∗ (1.0, 1.1)	1.05∗ (1.0, 1.1)	1.02 (.98, 1.1)	1.01 (.97, 1.1)
Pain duration (<3 years as reference)	1.51∗ (1.1, 2.0)	1.82∗∗ (1.3, 2.5)	1.15 (.85, 1.5)	1.34 (.96, 1.9)		
Leg pain (no as reference)	1.79∗∗ (1.3, 2.4)	2.10∗∗ (1.5, 3.0)	1.09 (.79, 1.5)	1.25 (.85, 1.9)		
Distal pain (no as reference)	1.27∗ (1.0, 1.5)	1.40∗ (1.2, 1.6)	1.07 (.72, 1.3)	1.20 (.85, 1.4)		
Psychological						
Anxiety (HADS)	1.06∗∗ (1.0, 1.1)	1.07∗∗ (1.0, 1.1)	.995 (.95, 1.0)	1.03 (.98, 1.1)		
Depression (HADS)	1.08∗∗ (1.1, 1.1)	1.09∗∗ (1.1, 1.1)	.989 (.94, 1.0)	.984 (.92, 1.1)		
TSK, Fear of movement	1.05∗∗ (1.0, 1.1)	1.05∗∗ (1.0, 1.1)	1.00 (.98, 1.0)	.992 (.96, 1.0)		
CSQ, Catastrophizing	1.05∗∗ (1.0, 1.1)	1.05∗∗ (1.0, 1.1)	1.00 (.98, 1.0)	1.01 (.98, 1.1)		
CSQ, Diversion	1.02∗ (1.0, 1.0)	1.02∗ (1.0, 1.0)	1.00 (.98, 1.0)	1.02 (.99, 1.1)		
CSQ, Reinterpretation	.995 (.98, 1.0)	.994 (.97, 1.0)				
CSQ, Cognitive coping	.974∗ (.95, .99)	.973∗ (.95, .99)	1.02 (.99, 1.1)	1.01 (.97, 1.1)		
IPQR, Symptoms	1.15∗∗ (1.1, 1.2)	1.16∗∗ (1.1, 1.2)	1.02 (.95, 1.1)	.981 (.90, 1.1)		
IPQR, Consequences	1.09∗∗ (1.1, 1.1)	1.09∗∗ (1.1, 1.1)	1.01 (.97, 1.1)	1.03 (.97, 1.1)		
IPQR, Cyclical time	1.00 (.96, 1.0)	1.01 (.97, 1.1)				
IPQR, Emotion representation	1.07∗∗ (1.1, 1.1)	1.06∗∗ (1.0, 1.1)	1.01 (.97, 1.1)	.952∗ (.91, .99)		.975 (.94, 1.0)
IPQR, Illness coherence	1.02 (.99, 1.0)	1.03∗ (1.0, 1.1)		1.01 (.98, 1.1)		
IPQR, Personal control	.932∗∗ (.90, .97)	.914∗∗ (.88, .95)	.974 (.93, 1.0)	.983 (.93, 1.0)		
IPQR, Treatment control	.930∗∗ (.90, .97)	.900∗∗ (.86, .94)	1.03 (.97, 1.1)	1.01 (.95, 1.1)		
IPQR, Timeline acute-chronic	1.07∗∗ (1.0, 1.1)	1.09∗∗ (1.1, 1.1)	1.04∗ (1.0, 1.1)	1.06∗ (1.0, 1.1)	1.04∗ (1.0, 1.1)	1.06∗∗ (1.0, 1.1)
IPQR, Psychological attributions	1.02 (.99, 1.1)	1.04 (1.0, 1.1)		1.02 (.96, 1.1)		
IPQR, Risk factors	1.01 (.97, 1.0)	1.02 (.99, 1.1)				
IPQR, Immunity	1.04 (.98, 1.1)	1.08∗ (1.0, 1.2)		1.00 (.90, 1.1)		
IPQR, Accident/chance	1.06 (.99, 1.1)	1.10∗ (1.0, 1.2)		.999 (.92, 1.1)		
Pain self-efficacy	.970∗∗ (.96, .98)	.967∗∗ (.96, .98)	.973∗ (.96, .99)	.973∗ (.96, .99)	.990 (.98, 1.0)	.989 (.97, 1.0)
Active coping	1.05 (.91, 1.2)	.996 (.85, 1.2)				
Passive coping	1.23∗∗ (1.1, 1.4)	1.31∗∗ (1.2, 1.5)	1.06 (.95, 1.2)	1.16∗ (1.0, 1.3)		1.11 (.98, 1.3)
Occupational						
Employed (reference)						
Employed, reduced duties (LBP)	1.86∗ (1.3, 2.6)	1.87∗ (1.2, 2.8)			1.13 (.75, 1.7)	1.26 (.78, 2.0)
Unemployed	1.89∗∗ (1.3, 2.7)	2.18∗∗ (1.5, 3.2)			1.15 (.75, 1.8)	1.37 (.84, 2.2)
Unemployed (LBP)	2.93∗∗ (2.0, 4.3)	3.86∗∗ (2.6, 5.8)			1.02 (.61, 1.7)	1.24 (.70, 2.2)

∗ *P* ≤ .05.

∗∗ *P* ≤ .001.

### Demographic Domain

Univariate tests show that age did not predict outcome at either 6 months or 5 years; female gender predicted poor outcome at 6 months but not at 5 years. Reporting having no further education beyond the age of 16 years and lower social class were each associated with an increased risk of poor outcome at 6 months and at 5 years. In the demographic multivariable model, only lower social class remained predictive (increased risk of clinically significant LBP at 6 months and at 5 years).

### Physical Domain

All variables in the physical domain significantly predicted risk of poor outcome in the univariate tests, but only pain intensity and disability remained after mutual adjustment for the intercorrelation between all the other variables within the pain domain model.

### Psychological Domain

Most of the variables within the psychological domain predicted clinically significant LBP status at 6 months and at 5 years. However, following adjustment in the psychological multivariable model, only a stronger baseline perception by the patient that his or her pain will last a long time (IPQR timeline acute-chronic variable) and lower pain self-efficacy (confidence in his or her ability to get on with life despite the pain) independently predicted clinically significant LBP at 6 months and at 5 years. A further 2 variables were predictive of the 5-year outcome but not the 6-month outcome: lower levels of emotional representations of one's back pain problem (IPQR–emotional representations) and higher passive coping.

### Occupational Domain

The occupational variable consisted of a single question, so no statistical adjustment was required. The model showed that compared to those in employment, persons on reduced duties because of their back problem at baseline, those who were unemployed, and those who were unemployed because of LBP were all at an increased risk of clinically significant LBP at 6 months and at 5 years.

### Final Multivariable Model

In the final multivariable model (Table 2), which combined significant factors remaining from each domain, baseline pain intensity significantly predicted 6-month outcome (RR = 1.12, 95% CI = 1.03–1.20) and showed a trend for 5-year outcome (RR = 1.09, 95% CI = .99–1.20), with a 12% and 9% increase in risk, respectively, per unit change on the pain intensity score. To give clinical understanding to this per unit change in score, a person with a one-third higher score of baseline pain intensity would have a 44% increase in risk of poor outcome at 6 months and a 33% increase in risk at 5 years. In addition, the IPQR timeline acute-chronic variable (ie, a measure of how long the person believes his or her back pain will last) showed a statistically significant increase in risk of 4% at 6 months (RR = 1.04, 95% CI = 1.01–1.07), and 6% at 5 years per unit change on the scale score (RR = 1.06, 95% CI = 1.03–1.09). Again to show clinical interpretation to this result, a person with a one-third higher score on the timeline scale at baseline would be at an increase of 32% in risk of poor outcome at 6 months and a 48% increase in risk at 5 years.

### Responder Nonresponder Analysis

Responders at 6 months were older (mean, 45.5 vs 42.2 years) and more likely to be female (61 vs 56%) than nonresponders (see Foster et al^10^ for full details of baseline and 6-month stages). Similarly, responders at 5 years were older (mean 47.4 vs 43.9 years), but there was no difference in gender. There were no reported differences between responders and nonresponders on the main outcomes (see Appendix 1 for full tests).

## Discussion

In a representative sample of UK primary care patients with LBP followed up for 5 years, elevated pain intensity around the time of the index healthcare visit and patients' perception that their back problems will last a long time were significant predictors of poor outcome in both the short and long term.

### Comparison With Existing Literature

This study confirms the findings from other prognostic studies that baseline pain intensity is a key predictor of future pain and disability status,^12^, ^24^ and it shows similar findings to other studies within primary care settings in respect to patient beliefs about the longevity of their LBP.^10^, ^16^ This study is the first to demonstrate this over a long time frame, and importantly shows the stability of these key prognostic factors over both the short- and long term. Two studies of primary care back pain patients, 1 of which combined baseline data from this cohort,^12^ showed that employment factors (eg, being unemployed, absence from work) predicted poor outcome at 12 months.^8^, ^12^ However, these studies utilized outcomes on the basis of disability,^12^ or a more severe level of pain and disability compared to this study,^8^ and it may be that employment factors play a greater role in predicting more severe outcome. One long-term study (Burton et al^4^) compared prognostic factors for LBP disability at 1 and 4 years. They report that fear avoidance, catastrophizing, and passive coping predicted poor outcome at 1 year but that depression and pain intensity predicted poor outcome at 4 years, and suggest that poor initial coping leads to longer-term depression. The current study does not reflect these findings, as there was little difference in factors predicting short-term (6 months) and long-term (5 years) outcome. The differences in findings between the current study and Burton et al might be explained by case mix and design. For example, the Burton et al study recruited patients seeking osteopathic treatment in private practice settings, with patients receiving at least 6 weeks of manipulative therapy after consultation. It may be that those who were initially depressed were less likely to respond to treatment, and the difference in the predictive factors for long-term disability may be an artifact of this response to treatment, rather than actual differences between individuals at baseline. One other study, albeit with only 12-month follow-up, that has design similarities to our study is done by Henschke et al.^16^ They studied primary care patients with LBP, included similar domain analysis, and used a measure similar to this study's timeline variable: a patient self-rated measure of how long his or her pain or disability will persist. They found that a patient's belief in the persistence of his or her back pain predicted poor outcome, alongside pain intensity, depression, and compensation claim status. Taken together with the current study's findings, this strengthens the conclusion that patients' beliefs about their back pain are important and robust prognostic markers in both the short and long term. A recent review on psychosocial risk factors for chronic LBP by Ramond et al^34^ reported that expectations about recovery conferred a more consistent risk factor than other psychological factors in predicting outcome; however, the included studies showing this effect had follow-up durations of up to 12 months. Our study has shown that such expectations are exerting a relatively strong influence (compared with a wide range of psychological and demographic factors) on long-term outcome 5 years later.

### Strengths and Weaknesses

Major strengths of this study are the large sample of primary care patients with LBP, long-term follow-up of 5 years, the comparison between predictive factors over the short and long term, and the wide range of predictors measured using validated instruments.^17^, ^24^ In addition, this study concurs with a comprehensive review of prognostic studies on the high level of chronicity over time^17^; however, we do acknowledge that a proportion of the baseline population would have changed in pain and disability status in the approximate 2 weeks between index consultation and response to baseline questionnaire, and this may have influenced the effect estimates.

Our results are bounded by the analysis model we have chosen. We chose a model that helped manage the large amount of predictor variables entered in the analysis by separating them into clinically meaningful domains of influence (ie, demographics, physical, psychological, occupational). This then allowed for us to select, statistically, the key predictor variables from each domain before moving them onto a final multivariable model. However, it is possible that predictor variables within one domain could have influenced the coefficient effects of another predictor variable in another domain had they been allowed to model together, for example, in a stepwise regression procedure. However, there are limitations with stepwise modeling; multiple testing may be increased (especially if the number of steps is large) and predictors would be selected solely on the condition of the previous step, which can lead to idiosyncratic models that are hard to replicate.^39^

Although we included a wide range of predictive factors, other factors not included here, such as length of unemployment and compensation status for LBP injury, have been shown to influence prognosis of LBP.^16^, ^38^ It may also be the case that predictors of LBP outcome are not fixed at specific time points (ie, at baseline in this study) but are more fluid in nature and change and evolve during different periods of the person's experience of LBP. Future studies with more frequent follow-ups will be better placed to identify such patterns. There was no evidence of bias caused by participant dropout on the prognostic factors or the outcome; therefore, our findings are unlikely to be substantially influenced by loss to follow-up.^10^, ^12^

### Clinical and Research Relevance

This study has shown that pain intensity predicts future pain and disability even after 5 years, and it confirms that pain relief is an important target not only in the initial management of the symptom but for the potential contribution to long-term improvement. Moreover, our results show that a strong personal belief that back pain will last a long time predicts clinically significant LBP independently of a wide range of other prognostic factors, including pain intensity, and additionally this belief appears to have enduring strength predicting both short- and long-term poor outcome. Collectively, the assessment of patients' pain intensity and this timeline belief could constitute important prognostic markers for the clinician, which could now be tested for their usefulness and impact in clinical practice. However, as outlined in the introduction of this paper, there is large variation within prognostic studies as to what prognostic factors are the most important. Consequently, this has led to debate and conflict between studies. Current thought suggests a more complex interaction between factors rather than singular predictive factors that influence the patient through time.^28^ In agreement, Ramond et al^34^ recently described psychosocial risk factors associated with LBP as momentary indicators that form part of a dynamic of the person in the context of his or her whole experience prior to, during, and after the pain episode. There is clearly a need now to investigate how prognostic factors work together, hypothesized and tested using more sophisticated techniques (eg, structured equation modeling), within longitudinal designs.

Although the identification of a prognostic marker is important to characterize those at higher risk, consideration should also be given to the potential for modification of such prognostic markers in the effort to alter patients' long-term outcome.^15^ Previously within this cohort, it was shown that change in the patient's global rating of his or her LBP was associated with change in the patient's timeline belief (ie, as pain reduced, so did the strength of belief of the patient that his or her back pain would last a long time), suggesting that a reduction in pain may lead to a reduction in the strength of the belief.^10^ Nevertheless, comparable belief constructs, such as a lower expectations about recovery (ie, the patient believes he or she will not get better), are associated with lower adherence to advice and treatment for LBP and may hinder recovery and maintain pain and disability.^23^ It may be that there is a reciprocal relationship between pain and the belief held by the patient, but clearly more research is required to investigate these interactions. However, there is evidence that such beliefs are modifiable.^13^ At present, the path of usual care for LBP (eg, NICE guidelines in the UK^36^) initially focuses on advice followed by physically based treatments, and not until later are psychological treatments advocated. Our findings add to those from other studies and indicate that a combined approach (ie, pain management and addressing patient's beliefs) early in the treatment process may be beneficial in averting a maladaptive and potentially harmful belief for patients with LBP.

### Conclusion

From a wide range of prognostic factors, the patient's baseline pain intensity and a belief that his or her LBP will last a long time increased the risk of clinically significant LBP in both the short and long term. Better pain management coupled with specific identification and modification of patients' perceptions of the future of their back problem are clear targets for clinical interventions.

## Figures and Tables

**Appendix Table 1a:** Test of Baseline Characteristics for Those Traced and Those Not Traced at Follow-Up

Variable	Traced (n = 698)				Not Traced (n = 112)				Significance
	Median	IQR	Mean	SD	Median	IQR	Mean	SD	
Age	47	14	46.27	9.42	41.5	19	41.42	11.12	<.001∗
RMDQ	8	9	8.72	5.79	8	8	8.90	5.52	.473
Pain intensity	3.67	3	3.94	2.27	4.33	3.08	4.27	2.41	.301
Depression	6	6	6.32	4.12	6	7	6.38	4.09	.715
Anxiety	8	6	8.10	4.42	8	6	8.45	4.43	.277
TSK	39	8	39.24	6.88	41	7.14	40.29	6.32	.204
Catastrophizing	8	10	9.34	7.47	9	10	10.40	7.89	.176
Diversion	16	11	15.48	7.97	16	14	15.94	8.72	.608
Reinterpretation	6	9	7.39	6.49	6	11	8.17	7.49	.551
Cognitive coping	17	8	16.77	6.18	16.50	9	16.64	6.37	.549
Symptoms	4	3	4.09	2.261	4	3	4.36	2.07	.130
Consequences	17	9	17.33	5.46	17	8	17.32	5.29	.818
Timeline cyclic	14	5	13.10	3.28	13.50	4.25	19.92	3.37	.751
Emotional	16	7	16.57	5.22	17	7.25	16.88	4.93	.483
Illness coherence	12	7	13.40	5.02	12	8.25	13.59	5.43	.477
Personal control	21	6	21.03	3.68	21.5	6	21.05	4.22	.542
Treatment control	17	5	17.20	3.23	17	4	16.84	3.57	.056
Timeline	21	9	19.77	5.79	21	9	20.17	6.18	.550
Attributions (psychological)	12	5	11.72	3.98	12	5	12.04	4.27	.536
Risk factors	15	6	14.94	4.01	15.5	5.55	15.11	4.08	.698
Immunity	6	3	5.22	1.91	6	3	5.19	1.82	.963
Accident	6	2	5.97	1.97	6	2	6.25	1.94	.337
Self efficacy	41	22.22	36.63	14.25	38	22	38.05	13.28	.482
Active coping	1	2	1.25	.92	1	1	1.25	.88	.851
Passive coping	2	2	2.18	1.36	2	2	2.35	1.31	.162
Categorical variables (Pearson chi-square tests)									
Gender									.342
Pain duration over 3 years									.772
Education level									.073
Social class									.178
Pain medication									.969
Consultation rate									.111

∗ Significant difference.

**Appendix Table 1b:** Test of Baseline Characteristics for Responders and Nonresponders

Variable	Responders (n = 488)				Nonresponders (n = 210)				Significance
	Median	IQR	Mean	SD	Median	IQR	Mean	SD	
Age	49	14	47.36	9.07	44	17	42.89	10.31	<.001∗
RMDQ	8	9	8.68	5.85	8	9	8.85	5.60	.242
Pain intensity	3.67	3	3.96	2.31	4	3.33	4.02	2.26	.558
Depression	6	6	6.26	4.23	6	6	6.43	3.94	.133
Anxiety	8	6	8.03	4.43	8	6.75	8.33	4.41	.323
TSK	39	8	39.09	6.92	40	7.56	39.84	6.63	.200
Catastrophizing	8	9.2	9.25	7.47	9	10.00	9.87	7.64	.145
Diversion	16	11	15.87	7.99	15	12	15.04	8.18	.041∗
Reinterpretation	6	9	7.39	6.52	6	10.75	7.65	6.81	.891
Cognitive coping	17	8	16.86	6.05	17	9	16.60	6.43	.361
Symptoms	4	3	4.11	2.25	4	4	4.16	2.21	.972
Consequences	17	8	17.29	5.45	17	8	17.38	5.41	.268
Timeline cyclic	14	5	13.00	3.25	14	5	13.19	3.35	.283
Emotional	16	7	16.55	5.27	17	7	16.72	5.05	.265
Illness coherence	12	7	13.13	4.94	13	8	13.89	5.25	.01∗
Personal control	21	6	21.17	3.72	21	5.75	20.83	3.81	.153
Treatment control	17	5	17.19	3.18	17	5	17.10	3.44	.983
Timeline	21	8	19.99	5.66	21	10	19.57	6.11	.386
Attributions (psych)	12	5	11.69	3.92	12	5	11.88	4.15	.575
Risk factors	15	6	15.02	3.99	15	6	14.88	4.07	.909
Immunity	6	3	5.22	1.88	6	3	5.19	1.91	.887
Accident	6	2	5.87	1.89	6	3	6.23	1.96	.04∗
Self-efficacy	41	21	39.10	14.02	40	23	37.69	14.22	.184
Active coping	1	2	1.23	.92	1	1	1.27	.91	.358
Passive coping	2	2	2.21	1.34	2	2	2.20	1.373	.668
Categorical variables (Pearson chi-square tests)									
Gender									.179
Pain duration over 3 years									.499
Education level									.649
Socioeconomic status									.703
Pain medication									.964
Consultation rate									.248

∗ Significant difference.

## Appendix 1

### Prognostic Indicators of Low Back Pain in Primary Care: 5-Year Prospective Study

Distributional checks and comparisons between those traced and not traced and between responders and nonresponders for the follow-up periods of 6 months and 5 years.

Distributional checks were carried out on continuous indicator variables to determine which showed non-normal distributions, indicating nonparametric testing for response and nonresponse and traced/nontraced groupings; chi-square testing was carried out for all categorical indicator variables. For differences between those who were traced (n = 698) and those not traced (n = 112) at the 5-year follow-up, analysis revealed a significant difference in age between those who were traced and those not traced. Those who were not traced were significantly younger in age (41.4 vs 46.3). There were no other significant differences found between traced and nontraced patients.

Comparing responders (n = 488) with nonresponders (n = 210) showed significant differences on age; nonresponders were significantly younger (43.3 vs 47.4). Nonresponders scored lower on the CSQ diversion score, higher on the IPQR illness coherence score and higher on the IPQR accident chance score. There were no other differences between responders and nonresponders.

Missing data analysis showed the measure of social class status to have missing data above 5% (actual 10.7%): data were more likely to be missing for those who were older in age, reported higher pain intensity and disability, greater depression, anxiety, catastrophizing, and fear avoidance and lower self-efficacy. Because of the significant differences for this missing group on these key variables and the risk of bias if completely removed from the analysis, it was decided to create a missing category group within the social class variable.^1^
